# Loss of independence: a novel but important global marker of illness

**DOI:** 10.1186/1757-7241-21-S2-A34

**Published:** 2013-09-09

**Authors:** Mikkel Brabrand, Jesper Hallas, Torben Knudsen

**Affiliations:** 1 Department of Medicine, Sydvestjysk Sygehus Esbjerg, Denmark; 2 Research Unit of Clinical Pharmacology, University of Southern Denmark, Denmark

## Background

As part of the assessment of all medical patients, vital signs are registered. However, each vital sign on its own (e.g. blood pressure, respiratory rate, pulse or peripheral oxygen saturation) only provide parts of the picture and are not considered global markers of illness. Loss of independence (LOI) (e.g. ability to stand, get into bed or out of a chair unaided) has been proposed as a global marker of illness. The present study present data on the association between LOI and 30-day mortality in acutely admitted medical patients.

## Methods

This was a prospective observational cohort study. Acutely admitted adult medical patients over a six months period at the medical admission unit at Sydvestjysk Sygehus Esbjerg were included. Upon arrival a nurse registered vital signs and LOI (defined as the ability to get into bed unaided). After inclusion of all patients, survival status was extracted from the Danish Civil Register. The association between LOI and 30-day mortality was assessed using both univariable analysis (Chi-square test) and multivariable logistic regression analysis controlling for the vital signs. Data will be presented as median (inter quartile range) or proportions.

## Results

A total of 5,894 patients were admitted (age 65 [49-77], 50.1% female) and 332 (5.6%) died within 30 days. LOI was reported on 5,064 patients (85.9%). Patients who had LOI had a significantly higher 30-day mortality, 16.7 vs. 2.0%, p < 0.001, Odds Ratio (OR) 9.63 (95% confidence interval [CI] 7.35-12.62). Patients with LOI were significantly older, had a lower systolic blood pressure and peripheral oxygen saturation and a higher pulse and respiratory rate and more patients had reduced level of consciousness. LOI had a sensitivity for 30-day mortality of 70.5% (95% CI 64.7-75.8), a specificity of 80.1% (79.0-81.3), positive predictive value of 16.7% (14.6-19.0) and a negative predictive value of 98.0% (97.5-98.4). In multivariable logistic regression analysis controlling for age, sex, systolic blood pressure, pulse, temperature, respiratory rate and peripheral oxygen saturation, LOI had an OR of 4.2 (95% CI 3.06-5.85), p < 0.001.

## Conclusion

LOI is a powerful marker of 30-day mortality of acutely admitted medical patients.

